# Call the Eckols: Present and Future Potential Cancer Therapies

**DOI:** 10.3390/md20060387

**Published:** 2022-06-09

**Authors:** Pedro Monteiro, Silvia Lomartire, João Cotas, João C. Marques, Leonel Pereira, Ana M. M. Gonçalves

**Affiliations:** 1 University of Coimbra, MARE—Marine and Environmental Sciences Centre, Department of Life Sciences, Calçada Martim de Freitas, 3000-456 Coimbra, Portugal; pmonteiro@student.uc.pt (P.M.); silvia.lomartire@uc.pt (S.L.); jcotas@uc.pt (J.C.); jcmimar@ci.uc.pt (J.C.M.); leonel.pereira@uc.pt (L.P.); 2 Department of Biology and CESAM, University of Aveiro, 3810-193 Aveiro, Portugal

**Keywords:** oncobiology, macroalgae, brown algae, phenolic compounds, phlorotannins, eckol-class compounds

## Abstract

In recent years, an increased interest in marine macroalgae bioactive compounds has been recorded due to their benefits to human health and welfare. Several of their bioactivities have been demonstrated, such as anti-inflammatory, antioxidant, anticarcinogenic, antibacterial and antiviral behavior. However, there still lacks a clear definition regarding how these compounds exert their bioactive properties. Of all the bioactive compounds derived from marine macroalgae, attention has been focused on phenolic compounds, specifically in phlorotannins, due to their potential for biomedical applications. Phlorotannins are a diverse and wide group of phenolic compounds, with several structural variations based on the monomer phloroglucinol. Among the diverse phlorotannin structures, the eckol-family of phlorotannins demonstrates remarkable bioactivity, notably their anti-tumoral properties. However, the molecular mechanisms by which this activity is achieved remain elusive and sparse. This review focuses on the described molecular mechanisms of anti-tumoral effects by the eckol family of compounds and the future prospects of these molecules for potential application in oncology therapies.

## 1. Introduction

Cancer, the uncontrollable growth of abnormal cells and dynamic alterations in the genome, causes cancerous features in normal cells. The progression of cancer impairs normal cell growth and processing, disrupting cellular metabolic “checkpoints”, eventually leading to what is established as “hallmarks of cancer”. These hallmarks are commonly known as universal characteristics of the pathology, such as sustained proliferative signaling and uncontrolled proliferation, evasion of growth suppressors, tissue invasion and metastasis, replicative immortality, promotion of angiogenesis and resistance to programmed cellular death [1]. Currently, it is known that several other factors contribute to the development of the pathology, such as the tumor microenvironment and the associated microbiota [2]. Since the discovery of the pathology, therapies and strategies for the treatment of cancer are constantly improving, with efforts in the discovery of new chemotherapies, new strategies for surgery, improved and focused radiotherapy and overall new insights and approaches to a disease as variable as the individuals that carry it.

Unfortunately, therapeutic approaches still come with risks to the patients: chemotherapies are often related to severe cytotoxicity [3]. Cancer therapy targets cancer cells, characterized by a high basal level of proliferation. Consequently, non-cancer cells with high proliferation rates are affected by the damaging effects of ionizing radiation and chemicals of traditional therapies, such as skin, hair and gastrointestinal epithelium. Other complications, such as nephrotoxicity and neurotoxicity, often arise due to the toxic effect of chemical medications, as anticancer toxic substances alter the normal activity of the physiological systems [4].

Even though there have been incredible advances in the last century of cancer combat, the paramount challenges remain undisputed, especially characteristics such as metastization, cancer cell stemness and pathology relapse, with cancerous cells displaying high levels of resistance to previously used chemotherapeutics [5,6]. Indeed, cancer resistance to chemotherapeutics is responsible for most of the failures in the treatment of the pathology. This resistance can be attributed to a myriad of factors, external or internal, in which the result is the inefficiency of the drug treatment. Therefore, there is high importance in finding new compounds that can be integrated into cancer treatments to ameliorate life quality. Although there is the availability of anti-neoplastic drugs and chemotherapy, the detrimental aftermath has sparked the search for natural products for potential use as new therapeutic agents [7]. In that context, natural products from the marine environment are considered to have enormous potential due to their bioavailability, specific and strong binding to drug targets and ability to bind to proteins with minimal entropy loss. Marine algae contain various phlorotannin derivatives, which are regarded as potent pharmacological polyphenols [8]. The increasing interest in macroalgae phenolic content research and therapeutic development has boosted the knowledge of possible bioactive compounds of interest that can be explored and developed. Among them, phlorotannins demonstrated bioactivity to cover a wide variety of ailments.

Phlorotannins are a unique set of compounds found in high concentration in brown macroalgae and in fewer amounts on red and green macroalgae and are structurally analogous to tannins from terrestrial plants. They are polymeric chains of phloroglucinol (1,3,5-tryhidroxybenzene) residues bound through C-C or C-O-C couplings. These compounds are highly hydrophilic, with molecular weights ranging from 160 Da to 650 kDa [5]. The biosynthetic pathway for the formation of phloroglucinol and consequently phlorotannin compounds are yet to be fully elucidated; however, it is assumed to occur via the condensation of acetate and malonate units through the shikimate or phenylpropanoid pathways. Presumably, two acetyl-CoA molecules in the presence of carbon dioxide are converted to malonyl-CoA. This process is catalyzed by a type III polyketide synthase, and the resultant chain undergoes cyclization and tautomerization, forming the end molecule phloroglucinol. Polymerization of the phloroglucinol moiety (through the above-mentioned C-C or C-O-C couplings) defines the structural variability characteristic of phlorotannins, which are further classified as fucols, phloroethols, fucophloroethol, eckols, fuhalols and carmalols [5,7]. Specifically, eckols contain an additional hydroxyl group on the terminal monomer and are characterized by the presence of 1,4-dibenzodioxin in their structure, exemplified by eckol, dieckol and 2-phloroeckol.

**Figure 1:**
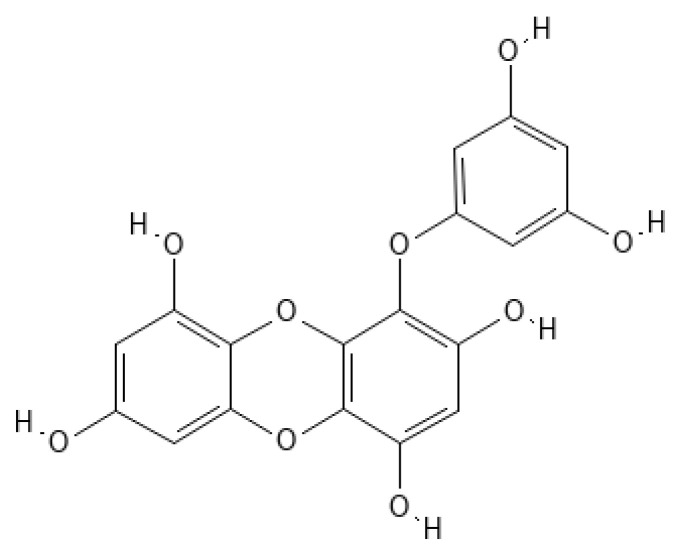
Eckol structure.

**Figure 2:**
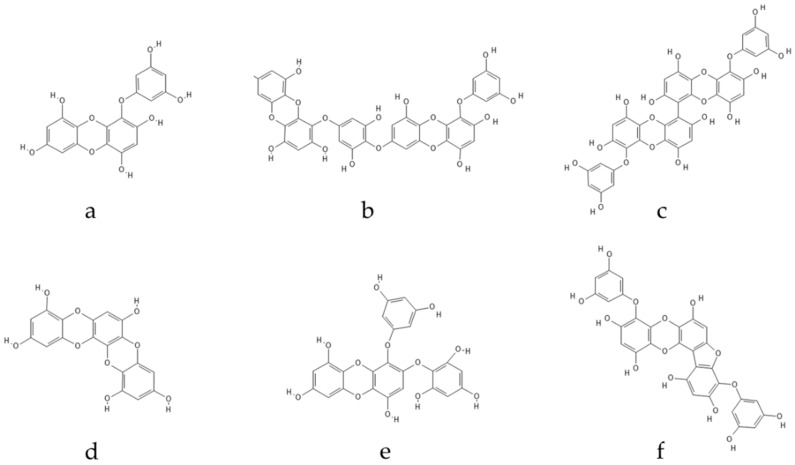
Eckol-class compounds: (**a**) Eckol; (**b**) Dieckol; (**c**) 6,6-Bieckol; (**d**) Dioxinodehydroeckol; (**e**) 2-phloroeckol; (**f**) Phlorofucofuroeckol.

Eckol (Figure 1) belongs to a class of phlorotannins, known to exist in high concentrations in the *Ecklonia* genus [7] but also found in other genera, such as *Fucus* or *Ascophyllum*. Eckol, a precursor compound illustrating the dibenzo-1,4-dioxin class of phlorotannins, contains phloroglucinol components linked to each other in multiple fashions, forming molecules such as dieckol, bieckol and phlorofurofucoeckol, among others (Figure 2). Eckol enriched and purified extracts have been shown to exhibit antioxidant, anti-inflammatory, hepatoprotective, neuroprotective, anti-obesity, anti-hypertensive, antibacterial, antiviral, anti-cancer and radioprotective activities [7]. Due to its numerous health properties, this compound has gathered much attention.

**Figure 3:**
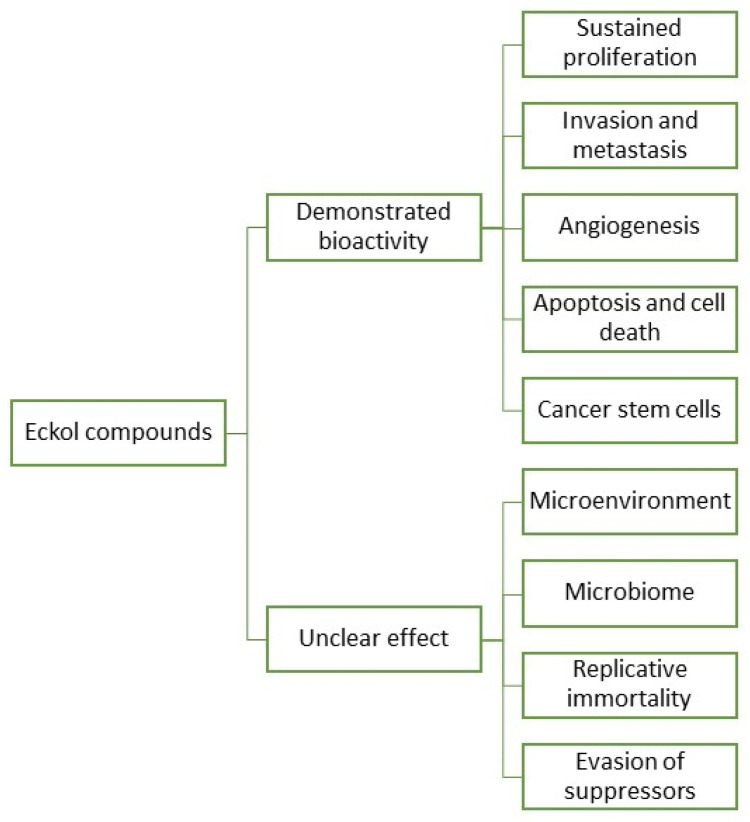
Eckol compounds demonstrated an unclear bioactivity against cancer hallmarks.

In fact, research and development for applications of eckols are gaining momentum, with a growing body of literature targeting the biotechnical applications of these compounds. However, information regarding eckols anti-cancer compounds is loose and spread, which makes it hard to infer their range of applications. As such, it is the objective of this review to cluster together the available information regarding eckols in the known hallmarks of cancer in order to enlighten the mechanisms of action by which these compounds exert their bioactive properties. Additionally, the future prospects of this class of compounds are also assessed, with insight into how other cancer hallmarks can be explored as targets for these compounds (Figure 3). There is mention of the interesting radioprotective effects of eckols with potential as therapeutic adjuvants, and finally, challenges in the extraction, purification and biological properties of eckols, such as their bioavailability and toxicity, are addressed.

## 2. Eckols as Potential Agents against Cancer Hallmarks

### 2.1. Eckols in Sustained Proliferation and Signaling

One of the most fundamental traits of cancer is sustained unregulated proliferation. In contrast to the careful regulation of growth and division in normal cells, cancer cells have unchecked production of growth signals, over-activating growth pathways. Generally, these alterations into prolific phenotypes occur through mutations in specific regulatory genes [1]. Cancer cells can sustain proliferative signaling in several ways. They produce growth factors themselves, to which they produce cognate receptors denominated autocrine proliferative stimulation, as well as signals to stimulate normal cells in the environment (tumor-associated stroma), which reciprocates through the production of growth factors for the cancer cell. Overproduction of surface receptors, conferring hypersensitivity of the cancer cell to a somewhat limiting factor of the milieu, was also reported [1,8,9]. There are several signaling pathways for growth and proliferation that can be altered into oncogenic mutations. Mitogen-activated protein kinase (MAPK) pathways are a conserved family of kinase modules that connect extracellular signals to intracellular machinery that regulates fundamental processes such as growth, proliferation, differentiation, migration and apoptosis [9]. The phosphatidylinositol 3-kinase/protein kinase-B/mechanistic target of the rapamycin (PI3K/AKT/mTOR) signaling pathway is a key intracellular pathway that regulates the survival, cell growth, differentiation, cellular metabolism and cytoskeletal reorganization of cells in reaction to a wide range of signals, including growth receptor tyrosine kinases (RTK) and G-protein coupled receptors [10]. NF-κB transcription factors are responsible for regulating the expression of key genes for innate and adaptive immunity, cell proliferation and survival and lymphoid organ development. NF-κB is found to be activated in many cancers by several stimuli, including pro-inflammatory cytokines such as IL-1β, epidermal growth factor (EGF), T-cells and B-cell mitogens, among others [11].

Some studies have demonstrated the potential effects of eckols as therapeutic agents against sustained proliferation (Figure 3 and Table 1). In a study by Zhang et al. [12], eckol was reported to inhibit the gene protein Reg3A, which induces the initiation, survival, growth and proliferation of pancreatic cancer cells. The human PaC cell line, SW1990, was used to test the antiproliferative effect of eckol. Cells were treated with 5, 10 or 20 µg/mL of eckol for 72 h. The treatment did not result in a significant change in cell viability for any concentration, but when cells were treated with Reg3A and eckol, results suggested that eckol did not have a significant direct cytotoxic effect on human SW1990 PaC cells but attenuated the Reg3A-mediated increase in SW1990 cell survival. Additionally, it appears that eckol reverted the Reg3A-mediated upregulation of JAK2, STAT3, NF-κB and cyclin D1, proteins involved in signaling pathways related to the proliferation, migration and apoptosis of cells, promoting a reduced proliferation of pancreatic cancer cells [12]. A reduced proliferation of cancer cell lines has been tested on the viability of three cellular lines (HeLa, H157 and MCF7) with compounds extracted from *Ecklonia maxima* (Phaeophyceae) [13]. Pretreated cells with eckol revealed a decrease in viability correlated with increasing eckol concentration, confirming the strong antiproliferative activity of eckol on HeLa, H157 and MCF7 cell lines. From the results, the authors believe that the high proliferation activity was due to cytotoxic activity of eckol against the cell lines, as the compound possesses functional hydroxyl groups that are well-positioned in the dibenzodioxin moiety that is effectively exposed to the cells and exert antiproliferative action. Dioxinodehydroeckol from *E. cava* has also been investigated against human breast cancer cells; the analysis confirmed the antiproliferative action through reduced expression of Bcl-2 anti-apoptotic proteins and NF-κB, triggered by the presence of dioxinodehydroeckol in tumoral cells. These pre-clinical studies suggest eckol class phlorotannins as potential candidates to perform clinical tests for breast cancer therapy [14]. Dieckol extracted from *Ecklonia cava* (Phaeophyceae) inhibited 12-O-tetradecanoylphorbol-13-acetate (TPA)-induced human hepatocellular carcinoma SK-Hep1 cell motility, and dieckol inhibited extracellular signal-regulated kinases 1/2 and c-Jun N-terminal kinases (JNKs), but not p38 Mitogen-Activated Protein Kinase (MAPK). TPA-induced activator protein-1 transcriptional activity was reduced by dieckol. Furthermore, dieckol administration significantly inhibited TPA-induced matrix metalloproteinase-9 (MMP-9) activity in SK-Hep1 cells. These findings demonstrate that dieckol acts as a powerful inhibitor of tumor promoter-mediated MAPK signaling pathways, resulting in the activation of Activator Protein 1 (AP-1) and MMP-9, and hence govern cancer cell motility [15]. In a study by Wang et al. [16], dieckol inhibited the proliferative and migratory properties of non-small–cell lung cancer cell line A549, interfering with Pi3K/AKT/mTOR signaling and caspases levels. It also induced cell apoptosis, mediating the increase of the tumor suppressor protein E-cadherin. Moreover, eckol assisted in the treatment of spheroid-forming glioma cells. Eckol successfully decreased sphere formation as well as CD133 cell population, blocking both Pi3K-AKT and Ras-Raf-1-Erk signaling pathways. It additionally suppressed the expression of glioma cell markers without causing cell death and significantly attenuated anchorage-independent growth on soft agar and tumor formation in xenograft mice. Importantly, eckol reduced the resistance of glioma-stem cells to ionizing radiation and temozolomide [17]. Furthermore, eckol suppresses stemness and malignancies, sensitizing glioma-stem-like cells to anticancer treatments, which can provide an alternative to current cancer treatments [18]. Eckol demonstrated the highest activity against HeLa cells, H157 and MCF7, which makes eckol a promising therapeutic candidate [13]. Overall, and concerning the traditional proliferative pathways that are known to be altered in cancer, eckols demonstrated potential activity as antiproliferative compounds, acting to some degree in signalization pathways, consequently halting the characteristic uncontrolled growth. It would be interesting to assess how and if eckols have any impact on the Wnt/β-catenin pathway. The Wnt/β-catenin signaling pathways are another cascade of signalization important in regulating cell proliferation, differentiation and migration. Reports demonstrate how this pathway is involved in several cancers (colorectal, breast), and reportedly, most alterations involve the stabilization of β-catenin, which is presumed to be correlated with tumor aggressiveness [11,19].

### 2.2. Eckols in Invasion and Metastasis

Cancer cells develop morphological alterations, as well as attachment modifications to other cells in the extracellular matrix (ECM). The best-characterized alteration in carcinoma cells is the loss of E-cadherin, a key adhesion molecule that forms adherens junction with neighboring cells. Increased expression of E-cadherin is well established as an antagonist of invasion and metastasis, and the reduction of E-cadherin expression levels is known to potentiate these phenotypes. The expression of genes encoding cell-to-ECM adhesion molecules is altered in aggressive carcinomas, while genes promoting cytostasis are often downregulated [32]. This stepwise invasion program is believed to be regulated by the epithelial-mesenchymal transition (EMT), prominently implicated as a means by which transformed epithelial cells can acquire the ability to invade, resist apoptosis and disseminate into other tissues [33,34]. This EMT program can be activated transiently or stably during the course of invasion and metastasis [7,20]. A set of transcription factors is believed to regulate EMT, such as Snail, Slug and Zeb1/2 [13]. Several of these factors can directly repress E-cadherin gene expression, allowing for the motility and invasiveness of cancer cells.

Dieckol derived from *E. cava* has been investigated in vitro against human fibrosarcoma cell invasion. Dieckol interfered with the migration and invasion of the HT1080 cell line by scavenging intracellular reactive oxygen species (ROS), which are also responsible for increasing migration and invasion of tumoral cells [25]. The inhibition of non-small–cell lung cancer cells migration and proliferation has also been detected in vitro using 6,6-bieckol from *Ecklonia cava* [20]. The compound inhibited the migration and induced apoptosis of tumoral cells, increasing the expression of E-cadherin and down-regulated Snail1 and Twist1 transcriptional levels, whose high expression is associated with lower survival rates in patients with cancer [35]. Lee et al. [21] tested both dieckol and phlorofucofuroeckol from *Ecklonia cava* to determine their antiproliferative and anti-invasive activity on human breast cancer MCF-7 and MDA-MB-231 cells. Dieckol and phlorofucofuroeckol reduced the migration and invasion of tumoral cells, as well as decreased the expression of receptor 4 (TLR-4) and NF-κB promoter-driven transcriptional activity, which is essential for cells proliferation, migration, tumor growth and inflammation. Dieckol reduced CoCl2-induced EMT in HT29 cells. Furthermore, dieckol therapy reduced ROS production, EMT marker protein expression and intracellular localization, cell motility and cell invasion. Dieckol may suppress hypoxia-induced EMT in HT29 cells via modulating cellular ROS and protein expression levels downstream of the HIF-1α signaling pathway, according to the findings of this study. Therefore, dieckol has the potential to be a promising therapeutic agent in the treatment of colorectal cancer [22]. Dieckol’s anticancer property against the non-small–cell lung cancer (NSCLC) cell line A549 is mediated by the inhibition of the invasive and migratory properties of A549 cells, as well as induction of apoptosis via inhibition of Pi3K/AKT/mTOR signaling and activation of the tumor suppressor protein E-cadherin, indicating that dieckol is a potent natural anticancer drug to treat NSCLC [36,37]. Dieckol extracted from *Ecklonia cava* inhibits Wiskott–Aldrich syndrome protein (WASP)-family verprolin-homologous protein 2 (WAVE2)-mediated invasive migration of B16 murine melanoma cells. The steady-state intracellular ROS levels in malignant B16F10 cells were greater than in parental, nonmetastatic B16F0 cells. H_2_O_2_ treatment boosted B16F0 cell migration and invasion capacity to levels comparable to B16F10 cells, suggesting that intracellular ROS signaling underlies the pro-metastatic features of B16 murine melanoma cells. ROS levels, as well as B16 melanoma cell motility and invasion capacity, were shown to be connected to Rac1 activation and WAVE2 expression. Overexpression of dominant negative Rac1 and siRNA-mediated WAVE2 depletion inhibited H_2_O_2_-induced cell invasion in B16F0 and B16F10 cells. Similarly, dieckol inhibits ROS-mediated Rac1 activation and WAVE2 expression, resulting in reduced B16 melanoma cell motility and invasion. Furthermore, we discovered that dieckol inhibits the interaction of WAVE2 with the NADPH oxidase component p47phox. As a result, the WAVE2 functions to link intracellular Rac1/ROS signaling to B16 melanoma cell invasive migration is blocked by dieckol [24]. Additionally, dieckol reduced MMP-2 and -9 expression in a dose-dependent manner, as well as cell invasion and cytomorphology in a 3D culture system on HT1080 cells. Furthermore, dieckol may influence the NF-κB route while having no effect on the activator protein-1 (AP-1) pathway or the tissue inhibitor of metalloproteinases (TIMPs). Finally, dieckol was able to drastically decrease MMP-2 and -9 expression as well as change the cytomorphology of the HT1080 cell line via the NF-κB pathway [38].

### 2.3. Eckols in Angiogenesis

During tumor progression, an angiogenic switch, which is turned transiently in adult individuals, is almost permanently activated, which causes vasculature to continuously sprout new blood vessels and sustain neoplasic growths. These angiogenic switches are regulated by a balance of factors that induce or suppress angiogenesis. A well-known angiogenic inducer is the vascular endothelial growth factor A (VEGF-A) [1,2].

The VEGF-A gene encodes ligands involved in forming new blood vessel growth during embryonic and postnatal development. VEGF-A signaling is modulated by three tyrosine kinases (VEGFR 1-3) and can be regulated both by hypoxia and oncogene signaling, thus sustaining tumor masses. Hypoxic conditions promote the expression of hypoxia-induced factors (HIF-1α) that lead to the expression of angiopoietin, matrix metalloproteinases (MMP’s) and VEGF, as well as other pro-angiogenic signals such as members of the fibroblast growth factor (FGF), that have been revealed to play a role in sustaining tumor angiogenesis when their expression is upregulated [39,40]. In some tumors, dominating oncogenes such as Ras and Myc can upregulate the expression of angiogenic factors, while in other cases, these factors are produced indirectly by immune cells.

A recent study by Shengtao Yang et al. [41] described how 7-phloro-eckol from *E. cava* demonstrated inhibition in pro-angiogenic factors in liver cancer, through analysis of the secretion of MMP-1, MMP-9 and VEGF proteins and HIF-1α under hypoxic conditions, in HepG2 and HUVEC cells. The results obtained demonstrated that 7PE could inhibit the production of HIF-1α under hypoxic conditions through the regulation of the MAPK and Pi3K pathways, consequently blocking the expression of VEGF. Saadeesh Kumar et al. [30] explored how the administration of dieckol would modulate the expression of pro-angiogenic factors (MMP, VEGF) in rats with N-nitrosodiethylmine (NDEA)-induced hepatocarcinogenesis. Their results demonstrated a reduction in the expression of MMP-2, MMP-9 and VEGF in NDEA rats treated with dieckol by more than 15% when compared to the expression in NDEA rats.

Overall, the literature supports the anti-angiogenic potential of eckols in hepatocellular cancer through the modulation of signaling pathways and factors in the development of new vasculature. It would be interesting to verify if the reduction in angiogenesis, demonstrated in hepatocarcinogenic models, is reflected in other cellular lines. One interesting observation could be that the direct regulation of angiogenesis by oncogenes that also drive proliferative signaling presumes that distinct tumor characteristics can be co-regulated by the same transforming agents [39].

### 2.4. Eckols in Resisting Programmed Cell Death (Apoptosis)

Programmed cell death by apoptosis serves as a natural barrier to cancer development. Apoptosis is triggered in response to physiological stresses cancer cells suffer during tumorigenesis and anticancer therapy [42]. The apoptotic “trigger” that regulates signals between regulators and effectors is controlled through a counterbalance between pro and anti-apoptotic members of the Bcl-2 family of regulatory proteins. Bcl-2 and its closest relatives (Bcl-xl, Bcl-2, Mcl-1 and A1) are apoptosis inhibitors that act through binding and suppressing two pro-apoptotic triggering proteins, Bax and Bak, that are embedded in the mitochondrial outer membrane. When these two pro-apoptotic proteins are relieved of inhibition by the Bcl-2 family, Bax and Bak disrupt the mitochondrial outer membrane, consequently releasing pro-apoptotic signaling proteins, in which the major player is cytochrome *c*. The released cytochrome *c* activates a cascade of caspases that, through their proteolytic activities, induce cellular changes associated with apoptosis [42].

Several abnormality sensors that play key roles in tumor development have been identified, notably the DNA-damage sensor that functions via the TP53 tumor suppressor. TP53 induces apoptosis by upregulating the expression of Noxa and Puma BH3-only proteins through response to the number of DNA damage and other chromosomal abnormalities. In contrast, insufficient factor signaling (e.g., low levels of interleukin-3 in lymphocytes or low insulin-like growth factor 1/2 [Igf1/2] in epithelial cells) can elicit apoptosis through a BH3-only protein called Bim. Furthermore, hyperactive signaling by oncoproteins such as Myc can trigger apoptosis, in part via Bim and other BH3 proteins [43].

Tumor cells acquire a variety of strategies to limit or evade apoptosis. Loss of the TP53 tumor suppressing function eliminates the damage-sensor from the apoptosis circuitry, and these ends can also be achieved by increasing the expression of anti-apoptotic regulators (Bcl-2) and survival signals (Igf 1/2), by downregulation of pro-apoptotic factors such as Bax, Bim and Puma, or by short-circuiting the extrinsic ligand-induced death pathway. The variety of apoptosis evading mechanisms can reflect the diversity of apoptosis-inducing signals that cancer-cell populations encounter in the evolution to the malignant state.

The therapeutic potential of dieckol against 7,12-dimethylbenz(a)anthracene (DMBA)-induced skin carcinogenesis (in mice) studies indicated that 30 mg/kg of dieckol supplementation significantly restored body and liver weight and reduced tumor incidence in DMBA-incited animals. In DMBA-induced rats, dieckol administration increased antioxidants (SOD, CAT, GPx and GSH) while decreasing phase-I enzymes Cyt-p450 and Cyt-b5. Dieckol also reduced pro-inflammatory modulators such as IL-6, IL-1 and TNF-α. Furthermore, the data showed that dieckol blocked the IB/NF-κB signaling pathway. Furthermore, dieckol increased the production of a pro-apoptotic protein (p53, Bax, caspase-3 and -9). Dieckol inhibited DMBA-induced skin cancer in mice, suggesting that it might be a promising agent [23]. Molecular pathways on dieckol have been explored for ovarian cancer cells (SKOV3 and A2780) subcutaneously inoculated in a xenograft mice model. Results evidenced that mice treated with dieckol have cytotoxic effects on A2780 and SKOV3 ovarian cancer cells, activate the expression of apoptotic proteins caspase-8, caspase-9 and caspase-3 and decrease the expression of AKT and p38, which are involved in cell apoptosis [29]. Dieckol also suppressed in vivo NDEA-initiated hepatocarcinogenesis by modulating xenobiotic-metabolizing enzymes, inducing apoptosis cascade and inhibiting proliferation, invasion and angiogenesis signaling [30]. Yoon et al. [28] investigated dieckol from *Ecklonia cava* subsp. *stolonifera* (formerly *Ecklonia stolonifera*) (Phaeophyceae) on human hepatocellular carcinoma (HCC) Hep3B cells. Results showed that cells treated with dieckol had reduced viability in a dose-dependent way; the presence of dieckol increased the permeability of mitochondrial membranes and the release of cytochrome *c* from mitochondria into the cytosol, which triggered apoptosis along with the proteins Bid, Bim, BAK, caspases-3, 7, 8 and 9 and cleaved poly (ADP-ribose) polymerase, all found in high expression in cells treated with dieckol. Thus, eckols demonstrate induction of apoptosis via the activation of both death receptor and mitochondrial-dependent pathways in tumoral Hep3B cells [28]. In vivo anti-tumoral effect of eckol has been examined in the development and progression of transplanted sarcoma in sarcoma 180 (S180) xenografts-bearing mice. Compared with the model control group, cells treated with eckol showed inhibitory tumoral effect at dosages of 0.5 and 1.0 mg/kg, revealing an inhibition by 36% and 52%, respectively, through apoptosis and antiproliferative activity, through modulation of the protein expression levels of Caspase-3 and Caspase-9 (two key apoptotic proteins) and Bcl-2 and Bax (two key anti-apoptosis-related genes), as well as epidermal growth factor receptor (EGFR, a proliferation stimulating protein). Results confirmed that eckol had no inhibitory effect on the weights of the thymus and spleen, whereas the spleen index of S180-bearing mice was significantly enhanced by 1.0 mg/kg eckol compared with the model control. It might suggest that eckol might have the potential to activate the anti-tumor immune response in vivo. A significant result is the stimulation of innate and adaptive immune responses in treated mice; activation of the mononuclear phagocytic system has been detected, along with major recruitment and activation of dendritic cells, which promoted the type 1 helper T cells anti-tumor response, increased the CD4+/CD8+ T lymphocyte ratio and enhanced cytotoxic T lymphocyte responses [26,27]. This pro-apoptotic capacity demonstrated by dieckol has also been detected in phlorofucofuroeckol A and dioxinodehydroeckol. The effect of phlorofucofuroeckol A has been investigated in human colorectal cancer (HT-29) cells for its anticancer property and results shown a high expression of activating transcription factor 3 (ATF3) which is associated with apoptosis in colorectal cancer. Therefore, this compound has been suggested as a candidate for the treatment of colorectal tumors, presumably through the capacity of phlorofucofuroeckol A to induce apoptosis via the ATF3-mediated pathway [31]. Dieckol increased intracellular ROS, and the antioxidant N-acetyl-L-cysteine (NAC) dramatically reduced dieckol-induced caspase activation, cytochrome *c* release, Bcl-2 downregulation, and apoptosis. Furthermore, dieckol suppressed AKT and p38 activity, and overexpression of AKT and p38, at least partially, restored dieckol-induced apoptosis in SKOV3 cells [29]. Overall, there is a large body of evidence that support the pro-apoptotic effects of eckols, with some mechanisms of action and inhibition demonstrated in vivo and in vitro.

### 2.5. Eckols in Cancer Cell Stemness

Cancer stem cells (CSC) are self-renewing cell populations that proliferate according to environmental stimulation and differentiate in heterogeneous tumor lineages [17]. However, and different from normal stem cells, there are, in fact, cancer-initiating cells and cancer-like stem cells, characterized by the clonal expansion that initiates and sustains tumor growth. These cells can originate from normal stem cells or progenitor cells after the acquisition of tumor initiating mutations. They are particularly important in the development of therapeutic resistance. While current radiotherapy and chemotherapy can remove the cancer tissue, cancer stem cells can survive in a transient, non-proliferative state and eventually resume growth later, promoting disease recurrence and metastasis, a process called minimal residual disease. Several mechanisms of drug resistance have been found in cancer stem cells, such as: (1) high expression of drug transporters of the ATP binding cassette (ABC) type, particularly ABC1/MDR1/P-gp and ABC2/BCRP, especially inside CSC populations; (2) highly efficient DNA repair; (3) resistance to committing programmed cell death (apoptosis); (4) quiescence and (5) decreased immunogenicity.

Tumor masses usually contain two types of CSCs: a small proportion that initiates and maintains malignant growth and differentiated progeny and a group of CSCs that remain dormant. In opposition to adult stem cells that divide asymmetrically and have a limited number of divisions, CSCs have symmetrical divisions, with progeny capable of unlimited replication. In case of therapeutic relapse, the dormant CSCs have a key role in resisting the therapeutic, acquiring resistance through selective pressure and adaptation, and finally activating and proliferating a resistant phenotype [1,7,30].

In a study by Hyun et al. [18], eckol was tested to inhibit the proliferation of cancer stem-like cells (CSCs) [44,45,46,47]. Sphere-forming glioma cells treated with eckol extracted from *Ecklonia cava* showed a reduction in self-renewal, sphere formation and anchorage-independent growth ability of glioma CSCs, acting on PI3K/AKT and RAF-1/ERK signaling pathways, which regulate the maintenance of CSCs. From the results, the antiproliferative activity emerged after treating CSCs with eckol concentrations from 50 to 90 μM. Eckol caused a reduction in the CD133+ cell population at 50 and 90 μM and it was also detected a decrease in CD133 expression, which is a marker associated with CSCs progression in many human cancer cell types. Therefore, 50 μM of eckol was considered by the authors as an optimum concentration to test the anti-tumoral activity in sphere-forming glioma cells. Moreover, eckol was investigated as an inhibitor of glioma stem-like cell populations. Sphere-cultured U373 glioma cells were dissociated into single cells and treated with eckol. Results showed that the treatment effectively suppresses self-renewal of glioma stem-like cells, as the expression levels of Sox2, Notch2 and β-catenin related to self-renewal were found to be down-regulated.

There is a relevant body of literature (Table 1) that supports the antiproliferative growth of CSCs through modulation of proliferative pathways such as MAPK and Pi3K, as well as reducing the expression of immunomodulatory factors that contribute to the growth and development of CSCs.

## 3. Cancer Hallmarks in Which Eckols Can Demonstrate Potential Bioactivities

### 3.1. The Stromal Role of the Microenvironment

Nowadays, it is well established that the biology of a tumor can only be understood by studying the individual cell types of each tumor, as well as the microenvironment constructed in the progression of the disease. Cancer cells initiate tumors, carrying the oncogenic and tumor suppressor mutations that define cancer as a genetic condition. However, the resulting heterogenic sub-clonal populations originated through hyperproliferation, and genetic instability confers a new dimension to cancer targeting and therapeutical approach. As such, many tumors are histo-pathologically diverse, containing different regions demarcated by various degrees of inflammation, differentiation, proliferation, vascularity and/or evasiveness [1,7]. These cancer cell populations reside within a complex multifactorial microenvironment composed of non-cellular ECM and cellular components. In the tissue niche, tumors exhibit a variety of cells that help drive tumor progression, just like an open system, where the tumor system interacts with the surrounding cells and alter the local environment.

It is believed that the extracellular matrix (ECM) is a key player in the progression of the tumor, providing both structural and biochemical support to the tumor cells [48]. Presumably, it is responsible for cell-to-cell communication, cell proliferation, cell motility and adhesion. Tumors frequently display desmoplasia, a fibrotic state characterized by the extensive deposition of ECM by cancer cells and carcinoma-associated fibroblasts. So far, the literature does not cover the capacity for eckols to modulate and influence the role of the microenvironment in which the tumor resides. Although there is some sparse information regarding the modulation and reduction of pro-inflammatory cytokines and other immunomodulatory factors that contribute to the progression of the tumor disease, as well as some influence in fibroblast growth factors and other components that have a great influence on the construction of the tumor ECM, the proper mechanisms of action by which eckols possess some degree of bioactivity is still unclear.

### 3.2. The Accessory Tumoral Microbiome

The microbiota has also been proposed to colonize tumors and presumably have systemic and local effects on cancer initiation, progression and treatment response. Reports have demonstrated that gut and intra-tumor microbiota can have an important role in modulating efficiency and response to chemo- and immunotherapeutic approaches [33].

It has been reported that Gammaproteobacteria, intra-tumor bacteria, can metabolize gemcitabine, a chemotherapeutic, into its inactive form, therefore granting resistance to the treatment [49]. In addition, Viaund et al. demonstrated that cyclophosphamide therapy induces the translocation of a specific set of Gram-positive bacteria from the small intestine to secondary lymphoid organs, prompting the generation of pathogenic T helper cells [37].

Furthermore, it was also demonstrated that a fungal community enriched in *Malassezia* spp., residing within pancreatic ductal adenocarcinoma in humans and mice, can also contribute to tumor progression [50], revealing how microbial communities residing within tumors are not necessarily homogeneous. While causal evidence between microbiota and carcinogenesis is still weak, increasing evidence suggests the microbiome is an integral part of the microenvironment of the tumor, with an impact on disease onset, progression and survival to anti-tumor therapies, contributing to tumor evolution [33].

A study by Nejman et al. [2] characterized the microbiome of 1526 samples from seven human tumor types. It was observed an association between different cancer types and specific microbiota and that certain tumor environments are enriched for common, relevant bacterial functional traits. The study validated the presence of bacteria in tumors and demonstrated their intracellular localization in both cancer and immune cells. The reason for bacterial colonization is not well defined. As tumor develops, the disorganized, leaky vasculature can allow circulating bacteria to enter the tumor microenvironment, finding refuge in the immunosuppressed tumor environment. There is also the possibility of intratumor bacteria arising from the adjacent tissues, which can be explained by the high similarity between the tumor microbiome and the natural adjacent tissues microbiome.

Regarding the microbiome and the potential influences of eckols, there are several reports that highlight the antimicrobial and antifouling properties of phlorotannins [51,52]. It would be interesting if the properties displayed by these compounds, specifically by eckols in antimicrobial assays and antifouling assays, could be extrapolated to the microbiota within tumor environments. Although several reports highlight that the bacteria within tumors are located intracellularly [2], to what extent are they only located within the tumor? Is there bacterial influence in the construction of the tumor microenvironment? More importantly, can this microbiota be targeted by eckols? Assessment of microbial communities and the possible influence of these compounds can help to further establish eckols as potential therapeutic agents, not only as agents that target the classical cancer hallmarks but as well as targeting the associated microbiota.

### 3.3. The Characteristic Replicative Immortality of Telomeres

Cancer cells require the unlimited replicative potential for the generation of macroscopic tumors, which contrasts with normal cell behaviour, that can only pass through a limited number of cell divisions. Cell division limitations are associated with two barriers to hyperproliferation: senescence and crisis/apoptosis. When cells are propagated in culture, they go through several cell divisions before senescence induction. Cells able to circumvent senescence usually go to the crisis phase, where most cells die. In the case of tumor cells, these phases are evaded and exhibit unlimited replication, a transition termed immortalization [1]. There are multiple lines of evidence pointing to the importance of telomeres and telomerase for enabling the unlimited replication capacity. Telomeres protect the ends of chromosomes, which shorten progressively upon cell division and eventually trigger cells to enter senescence or crisis [53], while telomerase is a specialized DNA polymerase that adds telomere repeats to the end of telomeric DNA, consequently increasing the viability of cellular divisions. Although telomerase is almost absent in non-immortalized cells, they are expressed at significant levels in the majority of immortalized cells. Consequently, the presence of telomerase is correlated with the resistance to senescence and crisis phases [1]. These two factors, senescence and crisis, function as fundamental barriers to excessive proliferation and are rationalized as crucial anticancer defenses. Immortalization of cells that eventually cause tumors has been attributed to their ability to maintain telomeric DNA length, avoiding senescence and apoptosis. This can be achieved by the upregulation of telomerase or, less frequently, a recombination telomere maintenance mechanism [1,24]. So far, there are no reports (that the authors had observed) that mention the capacity for eckols to regulate replicative immortality through the modulation of telomerase and telomeric DNA.

### 3.4. Evasion of Cellular Suppression Mechanisms

Cells must also circumvent programs that negatively regulate cell proliferation. The two prototypical tumor suppressors encode the RB (retinoblastoma associated) and TP53 proteins, which operate central control nodes within key complementary cellular circuits that govern the decisions of cells to proliferate or activate senescence and apoptosis [54]. While RB transduces growth inhibitory signals that originate outside the cell, TP53 receives input from stress and abnormality sensors that function with cells’ intracellular operating systems, such as damage to cells or levels of oxygenation, and others are suboptimal; TP53 can halt cell cycle progression. In cases where there is irreversible cellular damage, TP53 can trigger apoptosis [55].

Contact inhibition is also deactivated in cancer. Cell-to-cell contact formed by dense cell populations promotes growth suppression and inhibits further proliferation, while in tumorigenesis, normal tissue homeostasis is inhibited. The NF2 gene is implicated as a tumor suppressor as its loss triggers neurofibromatosis. The cytoplasmatic NF2 gene product Merlin regulates contact inhibition through the coupling of cell-surface adhesion molecules (e.g., E-cadherin) to transmembrane receptors tyrosine kinases (e.g., EGF receptor). Merlin enhances the adhesivity of cadherin-mediated cell-to-cell attachments, as well as sequestering growth factor receptors, efficiently limiting mitogenic signals [56].

Another mechanism of contact inhibition is through the LKB1 epithelial polarity protein, which organizes epithelial structure and maintains tissue integrity. LBK1 can regulate mitogenic signals from the Myc oncogene. When this LKB1 is suppressed, epithelial integrity is lost and becomes available for Myc-induced proliferation. LBK1 has also been identified as a tumor suppressor gene, lost in some human malignancies, reflecting the normal functioning of this protein as a suppressor of proliferation [57].

Another mechanism of suppressor evasion is through the corruption of the TGF-beta pathway, where in many late-stage tumors, this signaling pathway is redirected from cell suppressing to activation of epithelial-to-mesenchymal transition (EMT) consequently assisting in the invasion and metastasis of tumor cells [58].

To our knowledge, there is no reported effect of phlorotannins or eckols in the modulation of suppression evasion, as well as no information regarding any effects on the Myc oncogene, LBK1 and TGF-beta. Future studies can target these specific pathways and further verify the extent of eckols’ influence and bioactivity.

## 4. Future of Eckols as a Therapeutic

### Radioprotective Ability of Eckols

Radiotherapy is, alongside chemotherapy, the main treatment for cancer pathologies, with over 60% of cancer cases requiring radiation therapy [59]. In its essence, the radiation energy results in DNA damage in cancer cells, greatly limiting their proliferative potential. However, these treatments lack specificity, and it is common for neighboring non-cancerous cells to suffer the effects of radiation, in specific the normal cells that surround the tumor. As such, radiation therapy is frequently associated with side effects that compromise patients’ well-being, which has a toll on the mental and physical capacity for fighting the disease. As such, it is of interest the development of compounds or adjuvant therapy that can, while optimizing the damage to cancer cells, protect or relieve the radiation damage to non-cancerous cells. In that scenario, eckols have demonstrated interesting properties regarding their radioprotective bioactivities (Table 2).

In intestinal stem cells, eckol also demonstrated radioprotective activities. The pretreatment of gamma-irradiated mice with eckol demonstrated the survival and increased height of jejunal villi heights and an improvement of the jejunal crypt survival in comparison to untreated specimens. A decline in the number of apoptotic nuclei was observed 12 h after irradiation when pretreated with eckol. Eckol promoted protection against irradiation damage and shielding against apoptosis, promoting the survival of the jejunal crypts [66]. Additionally, the nuclei morphology of peripheral blood lymphocytes was assessed in irradiated mice, with and without pretreatment with eckol. Eckol increased the viability of lymphocytes, providing protection from radiation and inhibiting apoptotic events. Expression levels of p53 and Bax, commonly high after whole-body irradiation, were reduced after treatment with eckol, presumably due to the induction of Bcl-2, a pathway involved in apoptosis and DNA repair. Therefore, eckol administration can provide benefits for cancer treatments, providing protection to peripheral cells from irradiation damages. Additionally, nuclei morphology of peripheral blood lymphocytes was assessed in irradiated mice, with and without pretreatment with eckol. Eckol increased the viability of lymphocytes, providing protection from radiation and inhibiting apoptotic events. Expression levels of p53 and Bax, commonly high after whole-body irradiation, were reduced after treatment with eckol, presumably due to induction of Bcl-2, a pathway involved in apoptosis and DNA repair.

Eckol pretreatment efficiently scavenged ROS in gamma-irradiated V79-4 cells, protecting against DNA damage by decreasing oxidative damage. Eckol also demonstrated diminished lipid peroxidation and decreased apoptosis induction, with decreased DNA fragmentation. Additionally, an increase in Bcl-2 expression and reduction in Bax expression in irradiated cells after treatment with eckol demonstrated eckol ability to decrease apoptosis caspase-dependent pathway via mitochondria. Presumably, the major pathway suppressed by eckol in case of irradiation damage is SEK1-JNK-AP-1 [61].

Park et al. [60] investigated the radioprotective efficacy of eckol with the aim of proposing eckol as a candidate for adjuvant therapy to alleviate radiation-induced injuries to cancer patients. Eckol at dosages of 10 mg/kg body weight was administered to irradiated mice 2 h and 18 h before exposure. Results demonstrate that eckol can protect mice from radiation-induced damage compared with untreated mice. There is also the revival of hematopoietic ability from the splenic progenitor cells due to eckol’s protective ability, as well as a decrease of tail DNA length after eckol treatment in irradiation-damaged lymphocytes and a diminished death rate in comparison to the untreated group [63].

The radioprotective activity of eckol was also confirmed in another in vivo study [63], where the effect of eckol was investigated on lymphocytes and intestine against damage induced by single whole-body irradiation in mice. Eckol treatment modulated immunohistochemical location and amplitude of apoptosis response, protecting the lymphocyte’s viability and rescued intestinal cells from radiation-induced apoptosis by decreasing the amount of pro-apoptotic p53 and Bax and increasing the expression of antiapoptotic Bcl-2. Furthermore, the eckol radioprotective effect against γ-ray radiation-induced oxidative stress was investigated by Zhang et al. [61]. From the results, it has been confirmed that eckol pretreatment efficiently scavenged ROS in gamma-irradiated V79-4 cells, protecting against DNA damage by decreasing oxidative damage. Sadeeshkumar et al. [64] tested the radioprotective activity of dieckol in γ radiation-induced rat primary hepatocytes. Rat treated with dieckol showed reduced γ radiation-induced toxicity and enhanced the antioxidant activity, as well as decreased DNA damage and inflammation in hepatocyte cells. These results have been achieved due to the scavenging activity of dieckol on free radicals, already confirmed by Piao et al., where dieckol protected y-ray radiation-induced apoptosis of lung fibroblast cells by inhibiting ROS generation [65].Therefore, dieckol, as eckol, might be a potential aid for developing effective drugs to prevent antioxidant stress in patients under chemotherapy treatments.

Dioxinodehydroeckol inhibited the proliferation of MCF-7 cells with rates of 25, 40, 53, 56 and 64% at concentrations of 1, 5, 10, 50 and 100 µM, respectively, compared to the untreated control group. Presumably, the potential anti-proliferative activity of dioxinodehydroeckol lies in the ability to induce apoptosis through nuclear-factor-kappa-light-chain-enhanced activated B cells (NF-κB) family and NF-κB dependent pathway.

While there is substantiated evidence of the anti-cancer properties of phlorotannins in in vitro and in vivo assays, specific mechanisms of action and mechanisms of specific compounds remain entangled, with no evident separation of the wide family of compounds from the phlorotannin family and specific cell lines in which they exert bioactivities.

## 5. Bottlenecks in Eckols Implementation as a Therapeutic Alternative

### 5.1. Seasonal Variability of Macroalgae Bioactives

In nature, phenolic compound synthesis is often induced by extrinsic or intrinsic causes. In fundamental conformations, phenolic compounds (primary and secondary metabolites) have a natural and intrinsic beginning synthesis (primary metabolites) [67]. When seaweed cells are activated under stressful circumstances, they develop more complex forms. As a result, the presence of phenolic chemicals is invariably recognized in cells [68,69]. Extrinsic factors, on the other hand, cause cellular defensive reactions, which can modify the molecular process to create more and a wider variety of conformations of a given chemical class, particularly when it is a defensive component produced to defend algae from external aggressions [68].

There are inherent drivers in seaweed DNA and codifications that may inhibit natural phenolic compound synthesis. There are significant variations in the phenolic compounds generated by red, green and brown seaweeds, as well as their natural abundance [69,70] and intrinsic bioactivities. Species, life stage, size, age, thallus shape and reproductive status are also inherent variables [71,72]. Certain phenolic compounds must be produced at the cellular level. Phlorotannins, for example, need active phloroglucinol synthesis to construct phloroglucinol oligomers derivatives [62]. Extrinsic factors influencing the quality and quantity of seaweed phenolic compounds include seaweed geolocation, ecological characterization, season, biotic (herbivory or direct competition with other benthic organisms) and abiotic (salinity, pH, light incidence, temperature and water nutrient composition) factors [73,74].

### 5.2. Eckol Extraction and Isolation

Eckols can be extracted using standard solvent extraction procedures, such as methanol, ethanol, ethyl acetate, dichloromethane, butanol, water and acetone [75,76,77,78,79]. The solvent and kind of algae employed may have an effect on the total output of eckols extracted [76]. Furthermore, the solvent utilized in the extraction procedure can have a significant impact on the biological activity of the isolated phlorotannins. This is not only due to the fact that different solvents can vary the number of phenolic compounds extracted but also to the fact that different phlorotannins extracted according to solvent polarity have varying molecular weights and degrees of hydroxylation [77]. The purification of eckols, on the other hand, is a simple procedure that may be accomplished using silica gel thin-layer chromatography (TLC) [79,80], fast-centrifugal partition chromatography (FCPC) [78] and high-performance liquid chromatography (HPLC) [79].

Finally, the isolated eckols may be analyzed using several methods such as UV [75], 1H NMR [81] and ESI-TOF-MS spectrum analysis [82]. Dieckols UV–VIS absorption range had a peak between 230 and 290 nm [83].

### 5.3. Bioavailability

The major method examined for the administration of these compounds is the ingestion of polyphenols; hence, the biological activity of phlorotannins is regulated by their bioavailability, absorption, and metabolism. Due to a paucity of evidence on phlorotannin bioavailability, it is often assumed that this class of chemicals behaves similarly to other polyphenols found in terrestrial plants [84,85]. Due to the polymerized structure of phlorotannins, high molecular weight and interaction with other dietary constituents, studies have demonstrated that, like other tannins, the majority of non-absorbable dietary polyphenols will reach the colon intact [86]. The metabolism of phlorotannins in the body is thought to be exceedingly complicated, and the degree of biotransformation of these compounds during stomach digestion and intestinal fermentation processes will impact their biological effects in vivo [86]. Furthermore, the majority of the phlorotannins that enter the intestinal lumen intact, less than 15%, will be absorbed by the body. This suggests that the majority of these chemicals will be collected in the large intestine and contribute to the metabolic activities of the intestinal microbiota. As a result, increasing the stability of phlorotannins for in vivo applications is mandatory. For example, applying other compounds that further increase their stability and bioavailability or being transported by IV into the in vivo models [87]. Another important question regarding eckol bioavailability is the soluble properties of these molecules. Like most polyphenolic molecules, these are characterized by low solubility in water, which can be a challenge for the implementation of eckol compounds. Several studies demonstrate that the bioavailability of polyphenol can be improved through encapsulation of the molecules, increasing bioavailability and decreasing the rate of by-metabolizations [84,88,89] which crude extracts often encounter through administration.

### 5.4. In Vitro vs. In Vivo Questions on Eckols

As demonstrated above, eckols can play important roles as anticancer metabolites, acting in several stages of cancer progression such as proliferative signaling, metastasis, cell cycle, resistance to cell death, evasion, angiogenesis and growth suppressor evasion [90,91,92,93,94,95]. Eckols show cytotoxicity against many cancerogenic cell lines and the compounds’ selectivity towards cancer cell lines must be evaluated in hydrogel-based tumor models. These models provide the best real-world input for an anti-cancer treatment in the afflicted region, model, and imitate the dynamic tumor extracellular matrix [96,97]. This is one of the key issues with today’s anticancer medications [69]. As the compounds can have limited selectivity and can be counter-productive in their use, tests are required for secure anticancer compound accessibility. In this situation, chemical modification may be required to improve molecule selectivity, and more in vitro and in vivo research will be required before a commercial product is issued [69,70,94,95].

Thus, these questions are linked to promoting the applicability of eckols in future as anti-tumour drugs, and seaweed controlled cultivation can be a key to obtaining stable eckol molecules through a single extraction technique, guaranteeing high profile molecules that can be further studied and their biological properties further exploited [94].

### 5.5. Eckols Toxicity on Non-Cancerous Cells

Overall, the safety and toxicity of multiple phlorotannin compounds have been studied in human and animal cell lines [95]. Overall, most of the studies regarding phlorotannins toxicity have been performed on cell lines, and to our knowledge, four studies have assessed human toxicity [96,97,98,99]. Most of the studies demonstrate phlorotannins’ safety in human cell lines such as HeLa, HaCat, fibroblast, adenocarcinoma and melanoma cells. Of the eckol compounds, dieckol has been the most extensively studied and is reportedly non-toxic, but several reports have demonstrated mild toxicity according to the dosage delivered. Contrastingly, Ha et al. [76] showed that dieckol from *E. cava,* when administered at a dosage of 100 µg/mL, reduced the cell viability in haCat keratinocytes. Contrastingly, a study by Ko et al. [100] demonstrated that the administration of 100µg/mL of dieckol to zebrafish resulted in no associated toxicity, instead promoting cell viability. Yet, in another study by [101] in which the authors assessed the allergenic potential of crude phlorotannins, the authors reported mild toxicity for dieckol administered in a dosage of 500 µg/mL. Regarding the clinical trials, the reports support the absence of toxicity, as no side effects were detected in human trials with consumption of capsules with crude phlorotannins extracts with 250 mg/capsule/day. Nevertheless, 4 out of 20 humans subjects reported mild adverse effects. However, these symptoms did not deter to the volunteers to discontinue the trials [99,102]. Regarding other eckols, such as 6-6′-bieckol or 7-phloreckol, reports support to for of toxicity in cell lines [95]. Further studies and clinical trials must be performed to establish eckols as safe for consumption and to further develop possible therapies based on these compounds.

## 6. Conclusions

Eckols demonstrate remarkable properties in several characteristic “hallmarks of cancer” and possibly have the potential to modulate other mechanisms that have yet to be explored. Compounds from this family hold promise as a new therapeutic agents or as agents to be used as adjuvant in therapies due to their potent bioactivities. In addition to the anti-tumoral properties, the radioprotective properties of eckols hold promise as a future therapeutic adjuvant, which can provide a helpful boost to the demanding therapy that is radiotherapy. Although eckol and dieckol are the investigative focus of the eckol-family of compounds, 7-phloreckol and 6-6′-bieckol demonstrate interesting properties, and further exploration of these compounds can provide fruitful discoveries in the development of natural therapeutics against cancer. However, there are several challenges to the development of eckol-based therapies. Extractable biomass cultivation is difficult, technique standardization and extraction require optimization, in vivo testing is still in development and even when in vivo testing is fully permitted, challenges such as bioavailability, toxicity and drug design need to be assessed before the proper implementation and commercialization of these compounds as novel cancer therapeutics. Overall, it is necessary the leap from in vitro testing to in vivo, as the results obtained from testing in live models can further establish eckols as potential candidates for cancer therapy.

## Figures and Tables

**Table 1:** Anticancer activity of eckols.

Phlorotannin	Mechanism of Action	Anticancer Activity	Reference
Eckol	Interfere with Reg3A-mediated upregulation of JAK2, STAT3, NF-κB, cyclin D1 proteins	Antiproliferative action in pancreatic cancer cells	[12]
Eckol	-	Inhibitory activity against metastasis and reduced induced cell damages in cancer cell lines HeLa, H157 and MCF7	[13]
Dioxinodehydroeckol	Reduced expression of Bcl-2 and NF-κB proteins	Antiproliferative activity in human breast cancer cells	[14]
Dieckol	Inhibition of TPA-induced matrix metalloproteinase-9 (MMP-9) activity in SK-Hep1 cells	Control and regulation of cancer cell motility	[15]
Dieckol	Interfere with Pi3K/AKT/mTOR signaling and caspases level; increased expression of E-cadherin	Inhibition and apoptosis of non-small–cell lung cancer cell line A549	[16]
6,6-bieckol	Increase the expression of E-cadherin and down-regulated Snail1 and Twist1 transcriptional levels—associated with lower survival rates in patients with cancer	Inhibition of non-small–cell lung cancer cells migration and proliferation	[20]
Dieckol, phlorofucofuroeckol	Decrease in the expression of receptor4 (TLR-4) and NF-κB promoter-driven transcriptional activity	Reduced proliferation, migration, tumor growth and inflammation	[21]
Dieckol	EMT marker protein expression and intracellular localization, cell motility, and cell invasion suppress hypoxia-induced EMT in HT29 cells via modulating cellular ROS and protein expression levels downstream of the HIF1 signaling pathway	Reduced cell motility of colorectal cancer cells	[22]
Dieckol	Inhibition of Pi3K/AKT/mTOR signaling and activation of the tumor suppressor protein E-cadherin	Inhibition of Non-small–cell lung cancer A549 migration	[16]
Dieckol	Increase antioxidants (SOD, CAT, GPx, and GSH) while decreasing phase-I enzymes Cyt-p450 and Cyt-b5; reduce pro-inflammatory modulators such as IL-6, IL-1, and TNF-α	Inhibition of DMBA-induced skin cancer in mice	[23]
Dieckol	Inhibition of ROS-mediated Rac1 activation and reduce WAVE2 expression, which interacts with NADPH oxidase component p47phox	Reduction of B16 melanoma cell motility and blocked invasive migration	[24]
Dieckol	Inhibitor of MMP-2, -9 expressions by the downregulation of NF-κB pathway	Suppression of cell cancer invasion	[25]
Eckol	Modulation of caspase-3 and Caspase-9 expression; Bcl-2 and Bax; epidermal growth factor receptor	Apoptosis and antiproliferative activity	[26,27]
Dieckol	Regulation of Bid, Bim, BAK, caspases-3 -7 -8 -9, and cleaved poly (ADP-ribose) polymerase expression through the increase of permeability of mitochondrial membranes and the release of cytochrome c from mitochondria into the cytosol	Reduced cell viability in a dose-dependent way and apoptosis in tumoral Hep3B cells	[28]
Dieckol	Regulation of the expression of apoptotic proteins caspase-8, caspase-9, and caspase-3 and decreasing the expression of AKT and p38	Apoptotic effect on A2780 and SKOV3 ovarian cancer cells	[29]
dieckol	Suppression of NDEA-initiated hepatocarcinogenesis by modulating xenobiotic-metabolizing enzymes	Apoptosis and inhibited proliferation, invasion and angiogenesis signaling	[30]
phlorofucofuroeckol A	High expression of transcription factor 3 (ATF3), associated with apoptosis via the ATF3-mediated pathway	Apoptosis in colorectal cancer	[31]
Dieckol	Suppression of AKT and p38 activity; overexpression of AKT and p38	Apoptosis in SKOV3 cells	[29]
Eckol	Action on PI3K/AKT and RAF-1/ERK signaling pathways, which regulate the maintenance of CSCs	Inhibition of proliferation, reduction in self-renewal and anchorage-independent growth ability of glioma CSCs	[18]
Eckol	Blockage of both Pi3K/AKT and Ras/Raf-1/Erk signaling pathways	Suppression of expression of glioma cell markers avoiding cell death	[17]

**Table 2:** Radioprotective ability of eckols.

Phlorotannin	Mechanism of Action	Activity	Reference
Eckol	Revival of hematopoietic ability from the splenic progenitor cells	Photoprotective action in irradiation-damaged lymphocytes	[60]
Eckol	Regulation of pro-apoptotic p53 and Bax proteins; increased expression of antiapoptotic Bcl-2; increased height of jejunal villi and improvement of the jejunal crypt survival	Photoprotective action in intestinal stem cells for radiation-induced apoptosis	[61,62]
Eckol	Reduction of p53 and Bax proteins expression due to induction of Bcl-2	Photoprotective action to peripheral cells; increased viability of lymphocytes; inhibition of apoptotic events; DNA repair	[63]
Eckol	Increase in Bcl-2 expression and reduction in Bax expression; suppression of SEK1-JNK-AP-1 and caspase-dependent pathway via mitochondria	Decrease in lipid peroxidation and apoptosis-induction; decrease in DNA fragmentation	[61]
Dieckol	Scavenging activity on free radicals	Reduction in γ radiation-induced toxicity and enhanced antioxidant activity; decrease in DNA damage and inflammation in hepatocyte cells	[64]
Dieckol	Scavenging activity on free radicals	Apoptosis of lung fibroblast cells by inhibiting ROS generation	[65]

## Data Availability

Not applicable.

## References

[1] Hanahan D., Weinberg R.A. (2011). Hallmarks of Cancer: The next Generation. Cell.

[2] Nejman D., Livyatan I., Fuks G., Gavert N., Zwang Y., Geller L.T., Rotter-Maskowitz A., Weiser R., Mallel G., Gigi E. (2020). The Human Tumor Microbiome Is Composed of Tumor Type–Specific Intracellular Bacteria. Science.

[3] Nurgali K., Jagoe R.T., Abalo R. (2018). Editorial: Adverse Effects of Cancer Chemotherapy: Anything New to Improve Tolerance and Reduce Sequelae?. Front. Pharmacol..

[4] Karasawa T., Steyger P.S. (2015). An Integrated View of Cisplatin-Induced Nephrotoxicity and Ototoxicity. Toxicol. Lett..

[5] Shrestha S., Zhang W., Smid S.D. (2021). Phlorotannins: A Review on Biosynthesis, Chemistry and Bioactivity. Food Biosci..

[6] Vasan N., Baselga J., Hyman D.M. (2019). A View on Drug Resistance in Cancer. Nature.

[7] Manandhar B., Paudel P., Seong S.H., Jung H.A., Choi J.S. (2019). Characterizing Eckol as a Therapeutic Aid: A Systematic Review. Mar. Drugs.

[8] Karadeniz F., Kim S.-K. (2015). Antitumor and Antimetastatic Effects of Marine Algal Polyphenols. Handbook of Anticancer Drugs from Marine Origin.

[9] Dhillon A.S., Hagan S., Rath O., Kolch W. (2007). MAP Kinase Signalling Pathways in Cancer. Oncogene.

[10] Noorolyai S., Shajari N., Baghbani E., Sadreddini S., Baradaran B. (2019). The Relation between PI3K/AKT Signalling Pathway and Cancer. Gene.

[11] Feitelson M.A., Arzumanyan A., Kulathinal R.J., Blain S.W., Holcombe R.F., Mahajna J., Marino M., Martinez-Chantar M.L., Nawroth R., Sanchez-Garcia I. (2015). Sustained Proliferation in Cancer: Mechanisms and Novel Therapeutic Targets. Semin. Cancer Biol..

[12] Zhang M., Zhou W., Zhao S., Li S., Yan D., Wang J. (2019). Eckol Inhibits Reg3A-induced Proliferation of Human SW1990 Pancreatic Cancer Cells. Exp. Ther. Med..

[13] Mwangi H.M., Njue W.M., Onani M.O., Thovhoghi N., Mabusela W.T. (2017). Phlorotannins and a Sterol Isolated from a Brown Alga Ecklonia Maxima, and Their Cytotoxic Activity against Selected Cancer Cell Lines HeLa, H157 and MCF7. Interdiscip. J. Chem..

[14] Kong C.S., Kim J.A., Yoon N.Y., Kim S.K. (2009). Induction of Apoptosis by Phloroglucinol Derivative from Ecklonia Cava in MCF-7 Human Breast Cancer Cells. Food Chem. Toxicol..

[15] Oh S.-M. (2011). Dieckol Inhibits 12-O-Tetradecanoylphorbol-13-Acetate-Induced SK-Hep1 Human Hepatoma Cell Motility through Suppression of Matrix Metalloproteinase-9 Activity. J. Korean Soc. Appl. Biol. Chem..

[16] Wang C.H., Li X., Jin L., Zhao Y., Zhu G., Shen W. (2019). Dieckol Inhibits Non-Small-Cell Lung Cancer Cell Proliferation and Migration by Regulating the PI3K/AKT Signaling Pathway. J. Biochem. Mol. Toxicol..

[17] Efferth T. (2012). Stem Cells, Cancer Stem-like Cells, and Natural Products. Planta Med..

[18] Hyun K.-H., Yoon C.-H., Kim R.-K., Lim E.-J., An S., Park M.-J., Hyun J.-W., Suh Y., Kim M.-J., Lee S.-J. (2011). Eckol Suppresses Maintenance of Stemness and Malignancies in Glioma Stem-like Cells. Toxicol. Appl. Pharmacol..

[19] Mendoza M.C., Er E.E., Blenis J. (2011). The Ras-ERK and PI3K-MTOR Pathways: Cross-Talk and Compensation. Trends Biochem. Sci..

[20] Li Y., Liu M., Yang K., Tian J. (2021). 6,60-Bieckol Induces Apoptosis and Suppresses TGF-b-Induced Epithelial- Mesenchymal Transition in Non-Small Lung Cancer Cells. Chinese Herb. Med..

[21] Lee Y., Park J., Park S., Joo N., Lee B.H., Lee K.B., Oh S. (2019). Dieckol or Phlorofucofuroeckol Extracted from Ecklonia Cava Suppresses Lipopolysaccharide-Mediated Human Breast Cancer Cell Migration and Invasion. J. Appl. Phycol..

[22] Jeong S.-H., Jeon Y.-J., Park S.J. (2016). Inhibitory Effects of Dieckol on Hypoxia-Induced Epithelial-Mesenchymal Transition of HT29 Human Colorectal Cancer Cells. Mol. Med. Rep..

[23] Xiao W., Liu H., Lei Y., Gao H., Alahmadi T.A., Peng H., Chen W. (2021). Chemopreventive Effect of Dieckol against 7,12-dimethylbenz(a)Anthracene Induced Skin Carcinogenesis Model by Modulatory Influence on Biochemical and Antioxidant Biomarkers. Environ. Toxicol..

[24] Park S.J., Kim Y.T., Jeon Y.J. (2012). Antioxidant Dieckol Downregulates the Rac1/ROS Signaling Pathway and Inhibits Wiskott-Aldrich Syndrome Protein (WASP)-Family Verprolin-Homologous Protein 2 (WAVE2)-Mediated Invasive Migration of B16 Mouse Melanoma Cells. Mol. Cells.

[25] Park S.J., Jeon Y.J. (2012). Dieckol from Ecklonia Cava Suppresses the Migration and Invasion of HT1080 Cells by Inhibiting the Focal Adhesion Kinase Pathway Downstream of Rac1-ROS Signaling. Mol. Cells.

[26] Wang J., Zhang M., Zhao S., Liu J., Hu X. (2018). In Vivo Anti-Tumor Effect of Eckol, a Phlorotannin Component Isolated from Brown Algae, Associated with Regulating Dendritic Cells in Sarcoma 180 (S180) Xenografts-Bearing Mice. Proc. Annu. Meet. Jpn. Pharmacol. Soc..

[27] Zhang M.Y., Guo J., Hu X.M., Zhao S.Q., Li S.L., Wang J. (2019). An in Vivo Anti-Tumor Effect of Eckol from Marine Brown Algae by Improving the Immune Response. Food Funct..

[28] Yoon J.-S., Yadunandam A.K., Kim S.-J., Woo H.-C., Kim H.-R., Kim G.-D. (2012). Dieckol, Isolated from Ecklonia Stolonifera, Induces Apoptosis in Human Hepatocellular Carcinoma Hep3B Cells. J. Nat. Med..

[29] Ahn J.-H., Yang Y.-I., Lee K.-T., Choi J.-H. (2015). Dieckol, Isolated from the Edible Brown Algae Ecklonia Cava, Induces Apoptosis of Ovarian Cancer Cells and Inhibits Tumor Xenograft Growth. J. Cancer Res. Clin. Oncol..

[30] Sadeeshkumar V., Duraikannu A., Ravichandran S., Kodisundaram P., Fredrick W.S., Gobalakrishnan R. (2017). Modulatory Efficacy of Dieckol on Xenobiotic-Metabolizing Enzymes, Cell Proliferation, Apoptosis, Invasion and Angiogenesis during NDEA-Induced Rat Hepatocarcinogenesis. Mol. Cell. Biochem..

[31] Eo H.J., Kwon T., Park G.H., Song H.M., Lee S., Park N., Jeong J.B. (2016). In Vitro Anticancer Activity of Phlorofucofuroeckol A via Upregulation of Activating Transcription Factor 3 against Human Colorectal Cancer Cells. Mar. Drugs.

[32] Berx G., van Roy F. (2009). Involvement of Members of the Cadherin Superfamily in Cancer. Cold Spring Harb. Perspect. Biol..

[33] Micalizzi D.S., Farabaugh S.M., Ford H.L. (2010). Epithelial-Mesenchymal Transition in Cancer: Parallels Between Normal Development and Tumor Progression. J. Mammary Gland Biol. Neoplasia.

[34] Shlyakhtina Y., Moran K.L., Portal M.M. (2021). Genetic and Non-Genetic Mechanisms Underlying Cancer Evolution. Cancers.

[35] You J., Li M., Tan Y., Cao L., Gu Q., Yang H., Hu C. (2017). Snail1-Expressing Cancer-Associated Fibroblasts Induce Lung Cancer Cell Epithelial-Mesenchymal Transition through MiR-33b. Oncotarget.

[36] Talmadge J.E., Fidler I.J. (2010). AACR Centennial Series: The Biology of Cancer Metastasis: Historical Perspective. Cancer Res..

[37] Viaud S., Saccheri F., Mignot G., Yamazaki T., Daillère R., Hannani D., Enot D.P., Pfirschke C., Engblom C., Pittet M.J. (2013). The Intestinal Microbiota Modulates the Anticancer Immune Effects of Cyclophosphamide. Science.

[38] Zhang C., Li Y., Qian Z.-J., Lee S.-H., Li Y.-X., Kim S. (2011). Dieckol from Ecklonia Cava Regulates Invasion of Human Fibrosarcoma Cells and Modulates MMP-2 and MMP-9 Expression via NF-ΚB Pathway. Evid.-Based Complement. Altern. Med..

[39] Baeriswyl V., Christofori G. (2009). The Angiogenic Switch in Carcinogenesis. Semin. Cancer Biol..

[40] Ferrara N. (2009). Vascular Endothelial Growth Factor. Arterioscler. Thromb. Vasc. Biol..

[41] Yang S., Liu Y., Xiao Z., Tang Y., Hong P., Sun S., Zhou C., Qian Z.-J. (2021). Inhibition Effects of 7-Phloro-Eckol from Ecklonia Cava on Metastasis and Angiogenesis Induced by Hypoxia through Regulation of AKT/MTOR and ERK Signaling Pathways. Arab. J. Chem..

[42] Adams J.M., Cory S. (2007). The Bcl-2 Apoptotic Switch in Cancer Development and Therapy. Oncogene.

[43] Junttila M.R., Evan G.I. (2009). P53—A Jack of All Trades but Master of None. Nat. Rev. Cancer.

[44] Panchision D.M., McKay R.D.G. (2002). The Control of Neural Stem Cells by Morphogenic Signals. Curr. Opin. Genet. Dev..

[45] Singh S.K., Clarke I.D., Terasaki M., Bonn V.E., Hawkins C., Squire J., Dirks P.B. (2003). Identification of a Cancer Stem Cell in Human Brain Tumors. Cancer Res..

[46] Galli R., Binda E., Orfanelli U., Cipelletti B., Gritti A., De Vitis S., Fiocco R., Foroni C., Dimeco F., Vescovi A. (2004). Erratum: Isolation and Characterization of Tumorigenic, Stem-like Neural Precursors from Human Glioblastoma (Cancer Research (October 2004) 64 (7011–7021). Cancer Res..

[47] Wang S., Garcia A.J., Wu M., Lawson D.A., Witte O.N., Wu H. (2006). Pten Deletion Leads to the Expansion of a Prostatic Stem/Progenitor Cell Subpopulation and Tumor Initiation. Proc. Natl. Acad. Sci. USA.

[48] Bhowmick N.A., Neilson E.G., Moses H.L. (2004). Stromal Fibroblasts in Cancer Initiation and Progression. Nature.

[49] Geller L.T., Barzily-Rokni M., Danino T., Jonas O.H., Shental N., Nejman D., Gavert N., Zwang Y., Cooper Z.A., Shee K. (2017). Potential Role of Intratumor Bacteria in Mediating Tumor Resistance to the Chemotherapeutic Drug Gemcitabine. Science.

[50] Aykut B., Pushalkar S., Chen R., Li Q., Abengozar R., Kim J.I., Shadaloey S.A., Wu D., Preiss P., Verma N. (2019). The Fungal Mycobiome Promotes Pancreatic Oncogenesis via Activation of MBL. Nature.

[51] Kim H.J., Dasagrandhi C., Kim S.H., Kim B.G., Eom S.H., Kim Y.M. (2018). In Vitro Antibacterial Activity of Phlorotannins from Edible Brown Algae, Eisenia Bicyclis Against Streptomycin-Resistant Listeria Monocytogenes. Indian J. Microbiol..

[52] Pérez M.J., Falqué E., Domínguez H. (2016). Antimicrobial Action of Compounds from Marine Seaweed. Mar. Drugs.

[53] Blasco M.A. (2005). Telomeres and Human Disease: Ageing, Cancer and Beyond. Nat. Rev. Genet..

[54] Burkhart D.L., Sage J. (2008). Cellular Mechanisms of Tumour Suppression by the Retinoblastoma Gene. Nat. Rev. Cancer.

[55] Aubrey B.J., Strasser A., Kelly G.L. (2016). Tumor-Suppressor Functions of the TP53 Pathway. Cold Spring Harb. Perspect. Med..

[56] Curto M., Cole B.K., Lallemand D., Liu C.-H., McClatchey A.I. (2007). Contact-Dependent Inhibition of EGFR Signaling by Nf2/Merlin. J. Cell Biol..

[57] Partanen J.I., Nieminen A.I., Klefstrom J. (2009). 3D View to Tumor Suppression: Lkb1, Polarity and the Arrest of Oncogenic c-Myc. Cell Cycle.

[58] Ikushima H., Miyazono K. (2010). TGFβ Signalling: A Complex Web in Cancer Progression. Nat. Rev. Cancer.

[59] Mohan G., Ayisha Hamna T.P., Jijo A.J., Saradha Devi K.M., Narayanasamy A., Vellingiri B. (2019). Recent Advances in Radiotherapy and Its Associated Side Effects in Cancer—a Review. J. Basic Appl. Zool..

[60] Park E., Ahn G., Lee N.H., Kim J.M., Yun J.S., Hyun J.W., Jeon Y.J., Wie M.B., Lee Y.J., Park J.W. (2008). Radioprotective Properties of Eckol against Ionizing Radiation in Mice. FEBS Lett..

[61] Zhang R., Kang K.A., Piao M.J., Ko D.O., Wang Z.H., Lee I.K., Kim B.J., Jeong I.Y., Shin T., Park J.W. (2008). Eckol Protects V79-4 Lung Fibroblast Cells against γ-Ray Radiation-Induced Apoptosis via the Scavenging of Reactive Oxygen Species and Inhibiting of the c-Jun NH2-Terminal Kinase Pathway. Eur. J. Pharmacol..

[62] Cotas J., Leandro A., Monteiro P., Pacheco D., Figueirinha A., Gonçalves A.M.M., da Silva G.J., Pereira L. (2020). Seaweed Phenolics: From Extraction to Applications. Mar. Drugs.

[63] Park E., Lee N.H., Joo H.G., Jee Y. (2008). Modulation of Apoptosis of Eckol against Ionizing Radiation in Mice. Biochem. Biophys. Res. Commun..

[64] Sadeeshkumar V., Duraikannu A., Aishwarya T., Jayaram P., Ravichandran S., Ganeshamurthy R. (2019). Radioprotective Efficacy of Dieckol against Gamma Radiation-Induced Cellular Damage in Hepatocyte Cells. Naunyn. Schmiedebergs Arch. Pharmacol..

[65] Piao M.J., Kang K.A., Hyun J.W. (2009). Effect of Dieckol on Y-Ray Radiation-Induced V79-4 Lung Fibroblast Damage Involved in Modulation of Reactive Oxygen Species. J. Med. Life Sci..

[66] Moon C., Kim S.-H., Kim J.-C., Hyun J.W., Lee N.H., Park J.W., Shin T. (2008). Protective Effect of Phlorotannin Components Phloroglucinol and Eckol on Radiation-Induced Intestinal Injury in Mice. Phyther. Res..

[67] Lomartire S., Cotas J., Pacheco D., Marques J.C., Pereira L., Gonçalves A.M.M. (2021). Environmental Impact on Seaweed Phenolic Production and Activity: An Important Step for Compound Exploitation. Mar. Drugs.

[68] Domínguez H. (2013). Algae as a Source of Biologically Active Ingredients for the Formulation of Functional Foods and Nutraceuticals. Functional Ingredients from Algae for Foods and Nutraceuticals.

[69] Liu M., Hansen P.E., Lin X. (2011). Bromophenols in Marine Algae and Their Bioactivities. Mar. Drugs.

[70] Singh I.P., Sidana J. (2013). Phlorotannins. Functional Ingredients from Algae for Foods and Nutraceuticals.

[71] Wei R., Lee M.-S., Lee B., Oh C.-W., Choi C.-G., Kim H.-R. (2016). Isolation and Identification of Anti-Inflammatory Compounds from Ethyl Acetate Fraction of Ecklonia Stolonifera and Their Anti-Inflammatory Action. J. Appl. Phycol..

[72] Ahn G.-N., Kim K.-N., Cha S.-H., Song C.-B., Lee J., Heo M.-S., Yeo I.-K., Lee N.-H., Jee Y.-H., Kim J.-S. (2007). Antioxidant Activities of Phlorotannins Purified from Ecklonia Cava on Free Radical Scavenging Using ESR and H_2_O_2_-Mediated DNA Damage. Eur. Food Res. Technol..

[73] Kim A.-R., Shin T.-S., Lee M.-S., Park J.-Y., Park K.-E., Yoon N.-Y., Kim J.-S., Choi J.-S., Jang B.-C., Byun D.-S. (2009). Isolation and Identification of Phlorotannins from Ecklonia Stolonifera with Antioxidant and Anti-Inflammatory Properties. J. Agric. Food Chem..

[74] Jung H.A., Roy A., Jung J.H., Choi J.S. (2017). Evaluation of the Inhibitory Effects of Eckol and Dieckol Isolated from Edible Brown Alga Eisenia Bicyclis on Human Monoamine Oxidases A and B. Arch. Pharm. Res..

[75] Kim E.-A., Lee S.-H., Lee J.-H., Kang N., Oh J.-Y., Seun-heui S., Ahn G., Ko S.C., Fernando S.P., Kim S.-Y. (2016). A Marine Algal Polyphenol, Dieckol, Attenuates Blood Glucose Levels by Akt Pathway in Alloxan Induced Hyperglycemia Zebrafish Model. RSC Adv..

[76] Ha J.W., Song H., Hong S.S., Boo Y.C. (2019). Marine Alga Ecklonia Cava Extract and Dieckol Attenuate Prostaglandin E2 Production in HaCaT Keratinocytes Exposed to Airborne Particulate Matter. Antioxidants.

[77] Lopes G., Andrade P., Valentão P. (2016). Phlorotannins: Towards New Pharmacological Interventions for Diabetes Mellitus Type 2. Molecules.

[78] González-Colunga D., Antunes-Ricardo M., Gutiérrez-Uribe J.A., Cruz-Suárez L.E. (2019). Bioactivity-Guided Identification of Anti-AHPND (Acute Hepatopancreatic Necrosis Disease) Metabolites of Ecklonia Arborea. J. Appl. Phycol..

[79] Kim J., Yoon M., Yang H., Jo J., Han D., Jeon Y.-J., Cho S. (2014). Enrichment and Purification of Marine Polyphenol Phlorotannins Using Macroporous Adsorption Resins. Food Chem..

[80] Catarino M., Silva A., Cardoso S. (2017). Fucaceae: A Source of Bioactive Phlorotannins. Int. J. Mol. Sci..

[81] Kang M.-C., Kang S.-M., Ahn G., Kim K.-N., Kang N., Samarakoon K.W., Oh M.-C., Lee J.-S., Jeon Y.-J. (2013). Protective Effect of a Marine Polyphenol, Dieckol against Carbon Tetrachloride-Induced Acute Liver Damage in Mouse. Environ. Toxicol. Pharmacol..

[82] Yotsu-Yamashita M., Kondo S., Segawa S., Lin Y.-C., Toyohara H., Ito H., Konoki K., Cho Y., Uchida T. (2013). Isolation and Structural Determination of Two Novel Phlorotannins from the Brown Alga Ecklonia Kurome Okamura, and Their Radical Scavenging Activities. Mar. Drugs.

[83] Kim J.-H., Kim S.-B., Hwang H.-J., Kim Y.-M., Lee M.-S. (2016). Antibacterial Property of Ecklonia Cava Extract against Marine Bacterial Pathogens. J. Food Hyg. Saf..

[84] Munin A., Edwards-Lévy F. (2011). Encapsulation of Natural Polyphenolic Compounds; a Review. Pharmaceutics.

[85] Li Y.-X., Wijesekara I., Li Y., Kim S.-K. (2011). Phlorotannins as Bioactive Agents from Brown Algae. Process. Biochem..

[86] Sallam I.E., Abdelwareth A., Attia H., Aziz R.K., Homsi M.N., von Bergen M., Farag M.A. (2021). Effect of Gut Microbiota Biotransformation on Dietary Tannins and Human Health Implications. Microorganisms.

[87] Meng W., Mu T., Sun H., Garcia-Vaquero M. (2021). Phlorotannins: A Review of Extraction Methods, Structural Characteristics, Bioactivities, Bioavailability, and Future Trends. Algal Res..

[88] Grgić J., Šelo G., Planinić M., Tišma M., Bucić-Kojić A. (2020). Role of the Encapsulation in Bioavailability of Phenolic Compounds. Antioxidants.

[89] Rahaiee S., Assadpour E., Faridi Esfanjani A., Silva A.S., Jafari S.M. (2020). Application of Nano/Microencapsulated Phenolic Compounds against Cancer. Adv. Colloid Interface Sci..

[90] Wijesekara I., Yoon N.Y., Kim S.-K. (2010). Phlorotannins from Ecklonia Cava (Phaeophyceae): Biological Activities and Potential Health Benefits. BioFactors.

[91] Cotas J., Pacheco D., Gonçalves A.M.M., Silva P., Carvalho L.G., Pereira L. (2021). Seaweeds’ Nutraceutical and Biomedical Potential in Cancer Therapy: A Concise Review. J. Cancer Metastasis Treat..

[92] Catarino M., Silva A., Mateus N., Cardoso S. (2019). Optimization of Phlorotannins Extraction from Fucus Vesiculosus and Evaluation of Their Potential to Prevent Metabolic Disorders. Mar. Drugs.

[93] Hussain S.P., Hofseth L.J., Harris C.C. (2003). Radical Causes of Cancer. Nat. Rev. Cancer.

[94] Sepantafar M., Maheronnaghsh R., Mohammadi H., Radmanesh F., Hasani-sadrabadi M.M., Ebrahimi M., Baharvand H. (2017). Engineered Hydrogels in Cancer Therapy and Diagnosis. Trends Biotechnol..

[95] Negara B.F.S.P., Sohn J.H., Kim J.-S., Choi J.-S. (2021). Effects of Phlorotannins on Organisms: Focus on the Safety, Toxicity, and Availability of Phlorotannins. Foods.

[96] Shin H.-C., Kim S.H., Park Y., Lee B.H., Hwang H.J. (2012). Effects of 12-Week Oral Supplementation of Ecklonia Cava Polyphenols on Anthropometric and Blood Lipid Parameters in Overweight Korean Individuals: A Double-Blind Randomized Clinical Trial. Phyther. Res..

[97] Um M.Y., Kim J.Y., Han J.K., Kim J., Yang H., Yoon M., Kim J., Kang S.W., Cho S. (2018). Phlorotannin Supplement Decreases Wake after Sleep Onset in Adults with Self-Reported Sleep Disturbance: A Randomized, Controlled, Double-Blind Clinical and Polysomnographic Study. Phyther. Res..

[98] Baldrick F.R., McFadden K., Ibars M., Sung C., Moffatt T., Megarry K., Thomas K., Mitchell P., Wallace J.M.W., Pourshahidi L.K. (2018). Impact of a (Poly)Phenol-Rich Extract from the Brown Algae Ascophyllum Nodosum on DNA Damage and Antioxidant Activity in an Overweight or Obese Population: A Randomized Controlled Trial. Am. J. Clin. Nutr..

[99] Paradis M.-E., Couture P., Lamarche B. (2011). A Randomised Crossover Placebo-Controlled Trial Investigating the Effect of Brown Seaweed (Ascophyllum Nodosum and Fucus Vesiculosus) on Postchallenge Plasma Glucose and Insulin Levels in Men and Women. Appl. Physiol. Nutr. Metab..

[100] Ko S.-C., Cha S.-H., Heo S.-J., Lee S.-H., Kang S.-M., Jeon Y.-J. (2011). Protective Effect of Ecklonia Cava on UVB-Induced Oxidative Stress: In Vitro and in Vivo Zebrafish Model. J. Appl. Phycol..

[101] Le Q.-T., Li Y., Qian Z.-J., Kim M.-M., Kim S.-K. (2009). Inhibitory Effects of Polyphenols Isolated from Marine Alga Ecklonia Cava on Histamine Release. Process. Biochem..

[102] Okeke E.S., Nweze E.J., Chibuogwu C.C., Anaduaka E.G., Chukwudozie K.I., Ezeorba T.P.C. (2021). Aquatic Phlorotannins and Human Health: Bioavailability, Toxicity, and Future Prospects. Nat. Prod. Commun..

